# Editorial for Special Issue: “Synthesis and Application of Biomass-Derived Carbon-Based Nanomaterial”

**DOI:** 10.3390/nano13132020

**Published:** 2023-07-07

**Authors:** Mengmeng Zhang, Pengfei Li, Dapeng Wu

**Affiliations:** 1 Key Laboratory of Green Chemistry Medias and Reactions, Ministry of Education, School of Business, Henan Normal University, Xinxiang 453007, China; zhangmengmeng@htu.edu.cn (M.Z.); lipengfei@htu.edu.cn (P.L.); 2 School of Environment, Henan Normal University, Xinxiang 453007, China

Biomass-derived carbon-based nanomaterials represent a group of green and high-quality materials which can be potentially employed in the fields of environmental protection, energy conversion and clean energy storage. The unique composition and tissue structures of the biomass grant these carbon-based nanomaterials outstanding features, such as a high surface area, well-developed porous texture and active heteroatom doping sites. Meanwhile, the abundant sources of biomass further endows these materials with feasibility for potential large-scale applications, which has arouse great research interest from both the science and industrial communities. Our Special Issue consists of eight articles and two reviews, which are contributions to the topics of photothermal conversion, environmental protection, clean energy storage, and catalytic application. For example, Yeamsuksawat et al. prepared chitin-derived nitrogen-doped carbon nanopaper with subwavelength nano-porous structures for photothermal energy conversion. They found that its solar energy absorption could be improved by nitrogen doping, which greatly exceeds the performances of the commercial product [1]. 

In the field of environmental protection, Long et al. proposed a novel polyacrylic-acid-modified carbon skeleton derived from grapefruit peel, which could serve as solid-phase extraction coating to capture and determine volatile halogenated hydrocarbons in water. Due to their large specific surface area and abundant surface functional groups, the detection exhibits a wide linear range and good reproducibility [2]. In addition, Zhang et al. adopted Entermorpha prolifera (EP) as a precursor to prepare N–S co-doped bio-carbon with a layered porous structure and high specific surface area using a molten salt method to degrade sulfamethoxazole via persulfate activation. It was found that the performance of potassium-chloride-derived biochar was better than that of sodium-chloride-derived biochar, and the removal rate reached 99.6% in actual water treatment. Moreover, economic evaluation confirmed that this EP-derived biochar is more competitive than commercial activated carbon in cost, which demonstrates that the EP could be employed as an abundant biowaste that produces bio-carbons with high performances, and the molten salt strategy could be further optimized by adopting low-cost salt to reduce the cost of manufacture [3]. Based on this concept, Humulus scandens was firstly used as a biomass precursor to prepare biochar through a molten salt method by Bai et al. The as-derived biochar exhibited a high specific surface area, abundant oxygen-containing functional groups, and a good adsorption performance for heavy metal ions such as Cu^2+^ and Pb^2+^. In addition, the optimized biochar demonstrated good anti-interference ability and outstanding removal efficiency in simulated wastewater. The mechanism study and DFT calculation show that the oxygen functional group enhanced the binding energy of metal ions and plays a dominant role in the adsorption process [4].

Due to the well-developed porous structure and high conductivity as well as rich heteroatom doping sites, the biomass-derived carbons usually exhibit high performances in energy-storage devices. In this section, Wang et al. proposed a salt sealing technology combined with potassium hydroxide activation to convert pre-carbonized wheat shells into high-performance carbon materials. This novel method could achieve a good balance between a high surface area and mesoporous volume of biomass-derived porous carbon due to the molten salt and the activation of potassium hydroxide, which provide both channels for fast ion transfer and abundant active sites for charge storage [5]. Thomas et al. produced carbon particles from process lignin, sulfate lignin, soda lignin, lignin boost lignin and hydrolyzed lignin at different carbonization temperatures of 1000 °C and 1400 °C. It was found that the lignin source and carbonation temperature significantly affected the carbon quality and microstructure of carbon particles, which could in turn determine their performances in energy-storage devices [6]. To better illustrate the application of biomass-derived carbon materials in energy-storage devices, Ma et al. [7] and Yan et al. [8] comprehensively reviewed the recent progresses in the design, preparation and application of biomass carbon in the fields of lithium–sulfur batteries and sodium-ion energy-storage devices, respectively. 

The carbon materials with a highly porous structure as well as rich heteroatom doping sites usually possess high catalytic performances. For example, Wang et al. adopted a g-C_3_N_4_/carbon fiber composite as a carrier to immobilize lactase and glucose isomerase, which improved the efficiency of immobilized lactulose production. The g-C_3_N_4_/carbon fiber composite showed positive effects on the stability of the enzyme and endowed it with a high producing stability at a wide pH range [9]. In addition, Chen et al. adopted a supramolecular self-assemble strategy to synthesize supramylamine–cyanuric acid supramolecular aggregates. The as-fabricated carbon materials have an ordered layered microstructure, highly specific surface area, rich mesoporous distribution, and high N doping. In the Fenton system, the hydrogen peroxide production could be promoted, leading to the selective degradation of organic pollutants [10].

These works presented in this Special Issue demonstrated that the carbon-based materials prepared from biomass could not only pave a new way to yield high-performance materials for various applicational fields, but also contribute to the effective utilization of the abundant biomass resources. In addition, it was also proposed that bio-carbon manufacture techniques are of great significance to determine the performances of the final carbon materials. The further development of green, economic, sustainable and standardized techniques for the mass production of biomass carbon could provide more opportunities for the final commercialization of the biomass-derived carbon materials.
